# TRIM33 switches off *Ifnb1* gene transcription during the late phase of macrophage activation

**DOI:** 10.1038/ncomms9900

**Published:** 2015-11-23

**Authors:** Federica Ferri, Aude Parcelier, Vanessa Petit, Anne-Sophie Gallouet, Daniel Lewandowski, Marion Dalloz, Anita van den Heuvel, Petros Kolovos, Eric Soler, Mario Leonardo Squadrito, Michele De Palma, Irwin Davidson, Germain Rousselet, Paul-Henri Romeo

**Affiliations:** 1 CEA/DSV/iRCM/LRTS, 18 route du Panorama, Fontenay-aux-Roses 92265, France; 2 Inserm U967, Fontenay-aux-Roses 92265, France; 3 Université Paris-Diderot, Paris 75013, France; 4 Université Paris-Sud, Orsay 91400, France; 5 Equipe labellisée Ligue contre le Cancer, 18 route du Panorama, Fontenay-aux-Roses 92265, France; 6 Department of Functional Genomics and Cancer, Institut de Génétique et de Biologie Moléculaire et Cellulaire, CNRS/INSERM/ULP, BP 163, Strasbourg, Illkirch Cedex 67404, France; 7 Department of Cell Biology, Erasmus Medical Center, DR Molenwaterplein 50, Rotterdam 3015GE, The Netherlands; 8 The Swiss Institute for Experimental Cancer Research (ISREC), School of Life Sciences École Polytechnique Fédérale de Lausanne (EPFL), Lausanne CH-1015, Switzerland

## Abstract

Despite its importance during viral or bacterial infections, transcriptional regulation of the interferon-β gene (*Ifnb1*) in activated macrophages is only partially understood. Here we report that TRIM33 deficiency results in high, sustained expression of *Ifnb1* at late stages of toll-like receptor-mediated activation in macrophages but not in fibroblasts. In macrophages, TRIM33 is recruited by PU.1 to a conserved region, the *Ifnb1* Control Element (ICE), located 15 kb upstream of the *Ifnb1* transcription start site. ICE constitutively interacts with *Ifnb1* through a TRIM33-independent chromatin loop. At late phases of lipopolysaccharide activation of macrophages, TRIM33 is bound to ICE, regulates *Ifnb1* enhanceosome loading, controls *Ifnb1* chromatin structure and represses *Ifnb1* gene transcription by preventing recruitment of CBP/p300. These results characterize a previously unknown mechanism of macrophage-specific regulation of *Ifnb1* transcription whereby TRIM33 is critical for *Ifnb1* gene transcription shutdown.

## Abstract (web summary)

Transcriptional regulation of the interferon-β gene (*Ifnb1*) in macrophages is a critical immune event. Here, Ferri *et al*. show that, at late phases of macrophages activation, TRIM33 bound to a distal repressor element suppresses *Ifnb1* transcription by preventing recruitment of CBP/p300.

## Graphical abstract

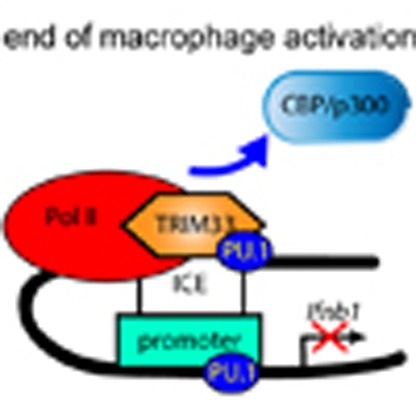

In response to viral or bacterial infections, germ line-encoded pattern recognition receptors induce type I interferon (IFN-I, encoded by 14 *Ifna* and 1 *Ifnb* genes in mice) gene transcription in myeloid and non-myeloid cells^1^. The secreted type I IFNs act early and transiently during the innate immune response to prime adaptive immunity and then type I *Ifn* gene transcription is shut down^2^. This transient expression of IFN-I is physiologically important. Whereas early IFN-I expression is important to control infection, pathogens that can overcome this initial control benefit from IFN-I mediated immune system suppression^3^,^4^.

Among type I *Ifn* genes, *Ifnb1* gene regulation has been a model system to study inducible transcription. After pattern recognition receptor-mediated activation, a group of transcription factors, including IRF3/IRF7, NF-κB and ATF2/c-jun, cooperatively binds to a regulatory sequence located between −102 and −47 bp upstream of the *Ifnb1* transcriptional start site (TSS) to form the *Ifnb1* enhanceosome. This enhanceosome recruits CBP/p300 for acetylation of histone H3 and permits recruitment of chromatin-remodeling complexes for initiation of transcription^5^.

Monocyte/macrophage-specific regulation of *Ifnb1* gene transcription has only been observed in the modulation of the transcriptional activity of IRF3. In monocytes, a PU.1-binding site located in the enhanceosome is constitutively bound by a IRF8/PU.1 myeloid complex that creates a preformed activation complex. This myeloid complex can recruit IRF3 through direct IRF3/IRF8 interaction and is involved in the rapid transcription of *Ifnb1* observed in monocytes after pathogenic stimulation^6^. Conversely, transcriptional regulator MafB can impair the interactions between IRF3 bound to the enhanceosome and co-activators, and thus negatively regulates *Ifnb1* transcription^7^. Finally, binding of transcriptional regulator YY1 to different sites within the enhanceosome has been shown to result in activation (at the beginning) or repression (at the end) of *Ifnb1* gene transcription in fibroblasts^8^,^9^,^10^. Altogether, these results point to the enhanceosome as a regulator of both initiation and termination of the *Ifnb1* transcription cycle and of monocyte/macrophage-specific regulation of *Ifnb1* transcription.

The tripartite motif (TRIM) protein family is characterized by a shared N-terminal structure consisting of a RING, two B-box domains, a coiled-coil domain and, for most of the TRIM family members, an E3 ubiquitin ligase activity^11^. TRIM33, together with TRIM24, TRIM28 and TRIM66, form a sub-family of TRIM proteins characterized by a C-terminal plant homeodomain juxtaposed to a bromodomain^12^. These TRIM proteins do not directly bind to DNA but can be recruited by DNA-binding proteins, such as nuclear receptors^13^, SMAD^14^, PU.1 or TAL-1 (refs 15, 16), to act as transcriptional regulators.

A large number of TRIM proteins are innate immune enhancers and act at multiple levels in signalling pathways, including production and action of IFN-β to confer antiviral and antibacterial cytokine production^17^,^18^. As for the TRIM33 sub-family, TRIM24 represses the viral-defense IFN response in hepatocytes as a consequence of repression of VL30 transcription^13^,^19^. Although these TRIM24 mediated repressions are potentiated by TRIM33, TRIM33 deficiency alone cannot elicit the IFN response in hepatocytes^13^,^19^. TRIM24 also acts as a negative regulator of the IFN/signal transducers and activators of the transcription signalling pathway^13^,^19^, and TRIM33 is essential for cytosolic RNA-induced NLRP3 inflammasome activation^20^. However, up to now, no TRIM protein has been shown to directly regulate *Ifnb1* gene transcription after recruitment to an *Ifnb1* regulatory sequence.

We have previously shown that TRIM33 can interact with PU.1 to repress its transcriptional activity during haematopoiesis and suggested that TRIM33 may regulate myeloid fate and have a role in macrophages^15^. Here, we show that TRIM33 deficiency in macrophages results in sustained expression of *Ifnb1* during the late phases of macrophages activation. Our results indicate that TRIM33, recruited by PU.1 on a distal regulatory element of the *Ifnb1* gene, regulates *Ifnb1* enhanceosome loading and shuts down *Ifnb1* gene transcription during the late stages of lipopolysaccharide (LPS) activation of macrophages by preventing recruitment of CBP/p300.

## Results

### TRIM33 controls *Ifnb1* expression in activated macrophages

**Figure 1:**
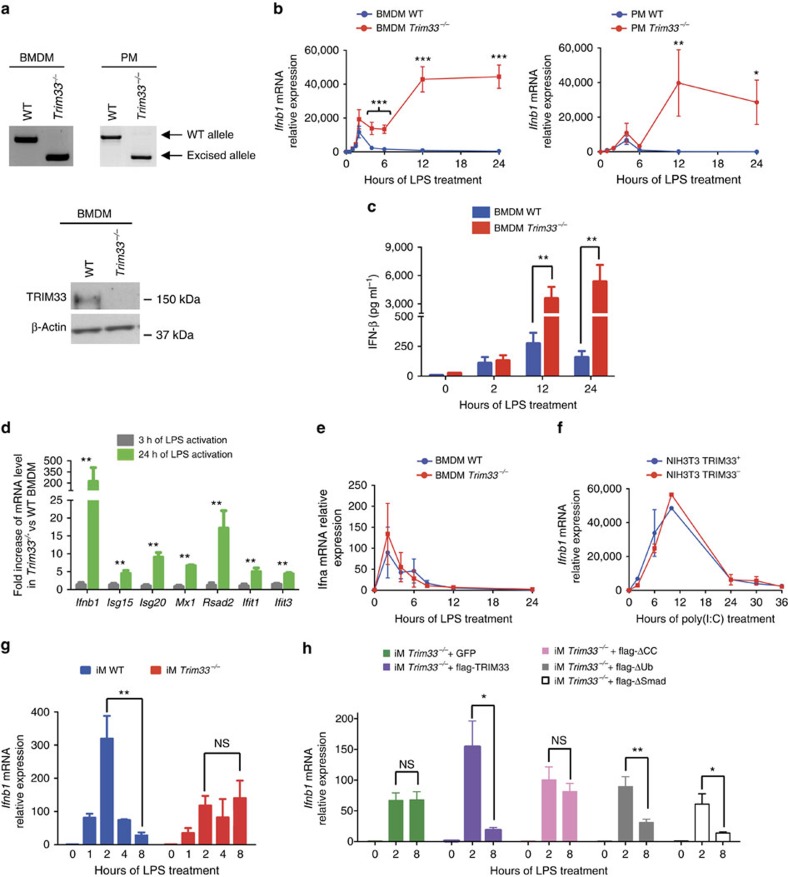
TRIM33 regulates *Ifnb1* gene expression in activated macrophages. (**a**) Genomic PCR detection of *Trim33* in BMDM and PM (top) and western blot analysis of TRIM33 in BMDM from WT and *Trim33*^*−/−*^ mice (bottom). (**b**) Kinetics of *Ifnb1* mRNA levels in WT and *Trim33*^−/−^ BMDM (left panel) or PM (right panel) after activation with LPS. Mean±s.e.m., *n*=3 to 6. (**c**) IFN-β protein levels in supernatants of non-activated and LPS-activated WT and *Trim33*^−/−^ BMDM. Mean±s.e.m., *n*=4. (**d**) Relative mRNA levels of *Ifnb1* and IFN-β target genes in *Trim33*^−/−^ BMDM versus WT BMDM treated with LPS (0.1 ng ml^−1^) for 3 or 24 h. Mean±s.e.m., *n*=3. (**e**) Kinetics of *Ifna* mRNA levels in WT and *Trim33*^−/−^ BMDM activated with LPS. Mean±s.e.m., *n*=3. (**f**) Kinetics of *Ifnb1* mRNA levels in NIH3T3 cells expressing a shRNA targeting *Trim33* (NIH3T3 TRIM33^*−*^) or luciferase (NIH3T3 TRIM33^+^) and activated with poly(I:C). Mean±s.e.m., *n*=3. (**g**) Kinetics of *Ifnb1* mRNA levels in WT and *Trim33*^−/−^ iM treated for the indicated times with LPS. Mean±s.e.m., *n*=3. (**h**) Relative expression of *Ifnb1* mRNA 0, 2 and 8 h after LPS treatment of *Trim33*^−/−^ iM transduced with a lentivirus coding for GFP (iM *Trim33*^−/−^ +GFP), for full-length flag-TRIM33 and GFP (iM *Trim33*^−/−^ +flag-TRIM33) or for flag-TRIM33 mutants lacking the coiled-coil domain (iM *Trim33*^−/−^ +flag-ΔCC), the ubiquitin ligase activity (iM *Trim33*^−/−^ +flag-ΔUb) or the SMAD-interaction domain (iM *Trim33*^−/−^ +flag-ΔSMAD). Mean±s.e.m., *n*=5. All qRT-PCR data are normalized to HPRT and, unless otherwise indicated, presented relative to expression of untreated WT cells. *P<0.05; **P<0.01 and ***P<0.001, Mann–Whitney test.

*Trim33*^*fl/fl*^ mice were crossed with mice expressing the Cre recombinase from the endogenous *Lysozyme2* locus (LyzCre) to generate *Trim33*^*−/−*^ mice. *Trim33*^*−/−*^ mice did not display developmental abnormalities and were healthy. PCR genotyping of purified haematopoietic cell populations showed deletion of *Trim33* in monocytes, bone-marrow-derived macrophages (BMDM), peritoneal macrophages (PMs) and neutrophils but not in myeloid progenitors, nor in B lymphocytes (Fig. 1a, upper panels and Supplementary Fig. 1a). The deletion of the *Trim33* gene allele was associated with a loss of TRIM33 expression in BMDM (Fig. 1a, lower panel).

The effects of *Trim33* deletion on *Ifnb1* expression in macrophages were studied by comparing kinetics of *Ifnb1* mRNA levels in wild-type (WT) or *Trim33*^*−/−*^ BMDM and PM, upon TLR4 activation by LPS or upon TLR3 activation by poly(I:C). These two activation protocols mimic bacterial (LPS) or viral (poly(I:C)) infections. A rapid early increase of *Ifnb1* mRNA levels was observed in WT and *Trim33*^*−/−*^ BMDM or PM activated by LPS (Fig. 1b). Thereafter, *Ifnb1* mRNA levels decreased from 2 h in WT BMDM (or 4 h in WT PM) and returned to basal levels by 6 h in WT BMDM and PM. In contrast, although *Ifnb1* mRNA levels decreased between 2 and 4 h, *Ifnb1* mRNA levels began to rise significantly by 6 h and remained high 24 h after LPS activation in *Trim33*^*−/−*^ BMDM or PM (Fig. 1b). This late sustained expression of *Ifnb1* was also observed when BMDM and PM were stimulated by poly(I:C) with *Ifnb1* mRNA levels being much higher at all late time points in poly(I:C) treated *Trim33*^*−/−*^ BMDM compared with WT (Supplementary Fig. 1b). The increased *Ifnb1* mRNA levels in *Trim33*^*−/−*^ BMDM activated with LPS correlated with increased levels of secreted IFN-β (Fig. 1c). Finally, using a sub-optimal concentration of LPS (0.1 ng ml^−1^) which did not activate *Ifnb1* in WT BMDM, we showed, in LPS-activated *Trim33*^*−/−*^ BMDM, an increased expression of genes whose transcription is dependent on IFN-β and occurs late during LPS activation (Fig. 1d). This increased expression was not accounted for by different responses of WT and *Trim33*^*−/−*^ BMDM to IFN-β (Supplementary Fig. 1c) and could be obtained after addition of IFN-β during the late phases of LPS activation of WT BMDM (Supplementary Fig. 1d). Altogether, these results show that TRIM33 deficiency is associated with increased *Ifnb1* mRNA level associated with IFN-β secretion during the late stages of LPS activation of BMDM.

To study if TRIM33 deficiency affects the expression of other *Ifn* genes, quantitative RT-PCR was performed and showed similar kinetics of *Ifna* mRNA levels in WT or *Trim33*^*−/−*^ BMDM with no late increased expression of *Ifna* mRNA levels in *Trim33*^*−/−*^ BMDM (Fig. 1e). Finally, highly efficient shRNA-mediated depletion of TRIM33 in NIH3T3 cells (NIH3T3 TRIM33^*−*^; Supplementary Fig. 1e) did not modify kinetics of *Ifnb1* mRNA levels in response to poly(I:C) (Fig. 1f), indicating cell-specificity in the regulation of *Ifnb1* expression by TRIM33.

Potential direct or indirect roles for TRIM33 in *Ifnb1* expression were addressed in ‘rescue' experiments in which TRIM33 protein was added back to LPS-activated immortalized *Trim33*^*−/−*^ macrophages (iM)^21^. As anticipated, *Trim33*^*−/−*^ iM bore the expected deletion of *Trim33* gene and these cells expressed no TRIM33 protein (Supplementary Fig. 1f). *Ifnb1* mRNA levels increased 1 h after LPS activation of WT and *Trim33*^*−/−*^ iM and peaked at 2 h with twofold higher observed expression in WT iM than in *Trim33*^*−/−*^ iM (Fig. 1g). As anticipated, *Ifnb1* mRNA levels in WT iM then dropped, whereas in *Trim33*^*−/−*^ iM, *Ifnb1* mRNA levels remained high (Fig. 1g). Thus, LPS activation of *Trim33*^*−/−*^ iM mimicked the late phase of the *Ifnb1* mRNA kinetics seen in *Trim33*^*−/−*^ BMDM. To address whether expression of ectopic TRIM33 could rescue *Ifnb1* downregulation at late time points, a flag-TRIM33-IRES-GFP (or GFP alone) construct was expressed in *Trim33*^*−/−*^ iM and cells were stimulated with LPS (Fig. 1h). The full-length exogenous TRIM33 protein restored *Ifnb1* downregulation at late time points of LPS activation of *Trim33*^*−/−*^ iM (Fig. 1h) indicating a role for TRIM33 in the regulation of *Ifnb1* gene expression during LPS activation of macrophage.

To determine the TRIM33 protein domains involved in *Ifnb1* regulation, mutant flag-TRIM33-IRES-GFP constructs lacking the coiled-coil domain (flag-ΔCC), the ubiquitin ligase activity (flag-ΔUb) or the Smad-interaction domain (flag-ΔSmad) were expressed in *Trim33*^*−/−*^ iM (Supplementary Fig. 1g). The flag-ΔUb and the flag-ΔSmad rescued normal *Ifnb1* expression at late time points of LPS-activated *Trim33*^*−/−*^ iM whereas the flag-ΔCC did not rescue normal profile of *Ifnb1* expression in activated *Trim33*^*−/−*^ iM (Fig. 1h). These results indicate that the Smad-interaction and ubiquitin ligase domains are not necessary for TRIM33 regulation of *Ifnb1* expression and suggest that TRIM33 is part of a regulatory complex that regulates *Ifnb1* transcription.

Altogether, these results reveal a role for TRIM33 in the regulation of *Ifnb1* expression specifically in downregulating its expression during the late stages of macrophage activation.

### TRIM33 binds a distal *Ifnb1* regulatory element

**Figure 2:**
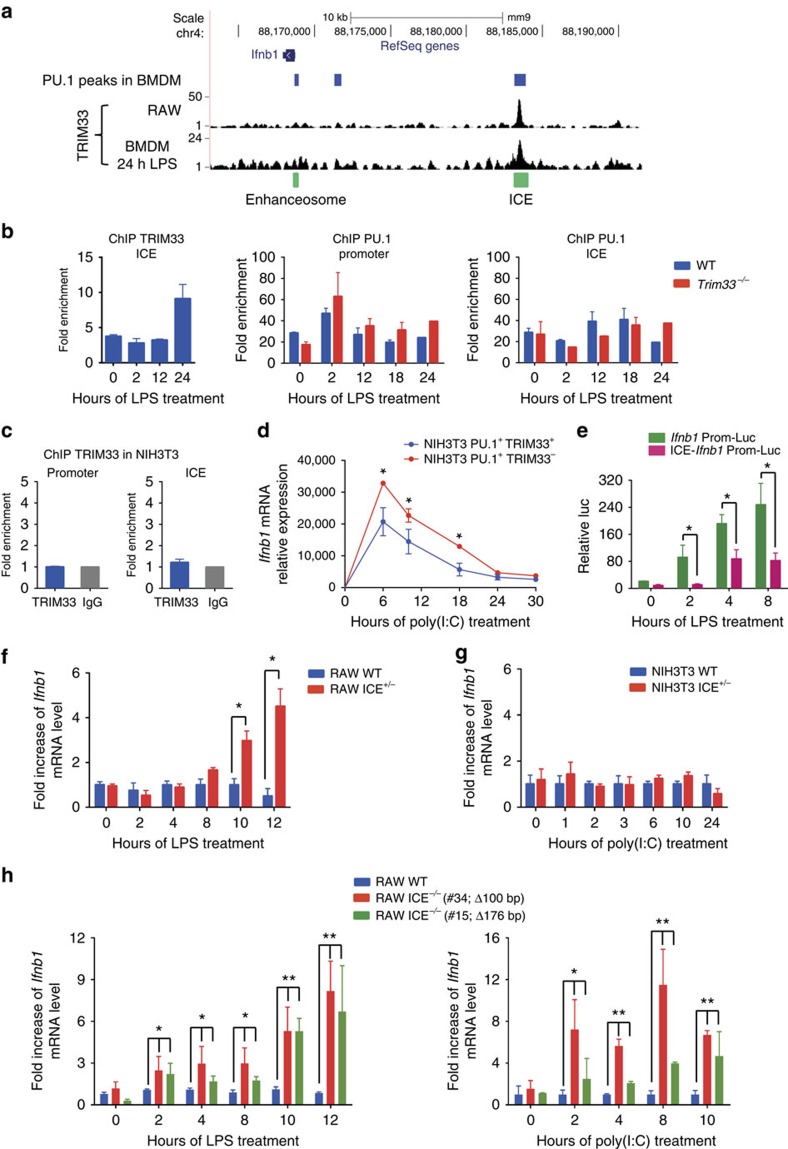
TRIM33 is bound to a distal *Ifnb1* gene regulatory element (ICE) in macrophages. (**a**) TRIM33 ChIP-seq data in RAW 264.7 cells and BMDM activated for 24 h with LPS showing TRIM33 binding to ICE, located 15 kb upstream the *Ifnb1* TSS. Blue boxes indicate PU.1 peaks in BMDM (data from ref. 43). Green boxes indicate positions of ICE and the enhanceosome. (**b**) ChIP-qPCR data for TRIM33 binding on ICE in WT BMDM (left panel) and for PU.1 binding on *Ifnb1* promoter and on ICE (right panels) in WT and *Trim33*^*−/−*^ BMDM at indicated time points after LPS activation. Data represent the enrichment over a negative control region in the *β-globin* promoter. Mean±s.e.m., *n*=3. (**c**) ChIP-qPCR analysis of TRIM33 binding at the *Ifnb1* promoter and on ICE in NIH3T3 cells. Mean±s.e.m., *n*=3. (**d**) Kinetics of *Ifnb1* mRNA levels in NIH3T3 cells expressing PU.1 and a shRNA targeting *Trim33* (NIH3T3 PU.1^+^ TRIM33^*−*^) or PU.1 and an shRNA targeting luciferase (NIH3T3 PU.1^+^ TRIM33^+^) and activated with poly(I:C). Mean±s.e.m., *n*=2. (**e**) Luciferase reporter assay in RAW 264.7 cells transfected with reporter constructs containing the *Ifnb1* promoter alone (*Ifnb1* Prom-luc) or ICE cloned upstream of the *Ifnb1* promoter (ICE-*Ifnb1* Prom-Luc) and activated with LPS. Mean±s.e.m., *n*=3. (**f**,**g**) Fold increase of *Ifnb1* mRNA levels in ICE^+/*−*^ versus WT RAW 264.7 cells after LPS activation (**f**) and in ICE^+/*−*^ versus WT NIH3T3 cells after poly(I:C) activation (**g**). Mean±s.e.m., *n*=3. (**h**) Fold increase of *Ifnb1* mRNA in ICE^−/−^ versus WT RAW 264.7 cells after LPS (left) or poly(I:C) (right) activation. Mean±s.e.m., *n*=3 to 5. Clones carrying different deletions at the PU.1/TRIM33 site in ICE are indicated in brackets. *P<0.05 and **P<0.01, Mann–Whitney test.

TRIM33 ChIP-seq data obtained from the RAW 264.7 murine macrophage cell line or from BMDM treated with LPS for 24 h showed a single TRIM33 peak at the *Ifnb1* locus that overlapped with a PU.1 peak located 15 kb upstream from the *Ifnb1* TSS (Fig. 2a). Interestingly, no TRIM33 occupancy was observed at the PU.1 peak located just upstream the *Ifnb1* promoter corresponding to the enhanceosome-binding sites. We called this −15 kb region the *Ifnb1* Control Element (ICE). Recently, the human homologue of ICE, named L2, has been shown to be a virus-inducible enhancer of *Ifnb1* in non-myeloid cells where it displays promoter and enhancer activities^22^.

Constitutive TRIM33 occupancy at ICE was observed in BMDM although occupancy appeared to increase at late time points (24 h) following LPS activation (Fig. 2b, left panel). PU.1 binding at the *Ifnb1* promoter and at ICE was constitutive and detected in WT and *Trim33*^*−/−*^ BMDM (Fig. 2b, middle and right panels) indicating that PU.1 binding to ICE was not TRIM33-dependent. Finally, no TRIM33 binding was observed on the *Ifnb1* promoter or ICE in NIH3T3 cells (Fig. 2c), indicating macrophage specificity of TRIM33 in binding to ICE.

To determine if TRIM33-mediated regulation of *Ifnb1* in myeloid cells can be obtained in NIH3T3 by simultaneous expression of PU.1 and TRIM33, NIH3T3 cells were transduced with a PU.1 expressing lentivirus (Supplementary Fig. 2a). PU.1 expressing NIH3T3 were then transduced with the lentivirus that promoted efficient shRNA-mediated depletion of TRIM33 (Supplementary Fig. 1e) and NIH3T3 PU.1^+^TRIM33^+^ and NIH3T3 PU.1^+^TRIM33^*−*^ responses to poly(I:C) were studied. Whereas TRIM33 deficiency did not modify kinetics of *Ifnb1* mRNA levels in response to poly(I:C) in normal NIH3T3 cells (Fig. 1f), TRIM33 deficiency increased *Ifnb1* mRNA levels in NIH3T3 PU.1^+^ cells treated with poly(I:C) but did not result in sustained expression of *Ifnb1* during the late stages of poly(I:C) activation (Fig. 2d). These results show that TRIM33 can repress *Ifnb1* expression in presence of PU.1 and indicate that additional factors that are not present in NIH3T3 cells are required for TRIM33-mediated late downregulation of *Ifnb1* expression.

To study the effect of ICE on *Ifnb1* promoter activity in myeloid cells, we used RAW 264.7 cells in which LPS regulates *Ifnb1* gene expression (Supplementary Fig. 2b). RAW 264.7 cells were transfected with luciferase reporter constructs containing a 391 bp fragment of the *Ifnb1* promoter preceded by a 408 bp fragment corresponding to the central region of ICE (ICE-*Ifnb1* Prom-Luc) or the fragment containing the *Ifnb1* promoter alone as a control sequence (*Ifnb1* Prom-Luc). Transcriptional activity of ICE-reporter (ICE-*Ifnb1* Prom-Luc) showed four to tenfold less activity than the promoter reporter in LPS-activated RAW 264.7 cells indicating that ICE functions as a cis-acting transcriptional repressor element in RAW 264.7 cells (Fig. 2e).

To study the role of ICE in RAW 264.7 and NIH3T3 cells, we used a CRISPR/Cas9 lentiviral system to deliver Cas9 and gRNA (Supplementary Fig. 2c). Using this system, we had to screen 200 RAW 264.7 clones to get one RAW 264.7 clone with monoallelic deletion of ICE (Supplementary Fig. 2d). This low efficiency of recombination in RAW 264.7 cells does not favour the use of transient transfection for CRISPR/Cas9-mediated deletion of ICE, transient transfection efficiency in RAW 264.7 being 15% compared with 90% using the lentiviral system. RAW 264.7 and NIH3T3 clones bearing this deletion on one allele (termed RAW ICE^+/*−*^ and NIH3T3 ICE^+/*−*^, respectively; Supplementary Fig. 2d) were analysed for *Ifnb1* expression after LPS or poly(I:C) activation. In LPS-activated RAW WT cells, *Ifnb1* mRNA levels increased during the first 4 h and then gradually decreased (Supplementary Fig. 2e). In contrast, whereas *Ifnb1* mRNA levels increased comparably in RAW ICE^+/*−*^ cells, they subsequently remained stable (Fig. 2f and Supplementary Fig. 2e). In NIH3T3 WT and ICE^+/*−*^ cells activated with poly(I:C), kinetics of *Ifnb1* mRNA levels were similar (Fig. 2g and Supplementary Fig. 2f). These results indicate that ICE plays a role in the negative regulation of *Ifnb1* expression at late stages of LPS activation in RAW cells.

Sequential deletions centered on the PU.1/TRIM33-binding site of ICE were performed in RAW 264.7 cells using CRISPR/Cas9 technology (Supplementary Fig. 2g). Of the 96 clones screened, two carried a biallelic deletion of ICE (Supplementary Fig. 2d). Two RAW 264.7 clones bearing a 100 or 176 bp deletion on the two *Ifnb1* alleles (termed #34 and #15; Supplementary Fig. 2g) were analysed for *Ifnb1* expression after LPS or poly(I:C) activation. In the two clones high and sustained *Ifnb1* mRNA levels were found but only after LPS or poly(I:C) activation, with the highest increase being obtained during the late phase of activation (Fig. 2h).

Altogether, these results show that ICE functions as a cis-acting transcriptional repressor element of the *Ifnb1* gene activation in macrophages and indicate that TRIM33 and PU.1 bound to ICE may have an important role in *Ifnb1* gene transcription shutdown during the late phase of macrophage activation.

### ICE chromatin structure and interaction with *Ifnb1* in BMDM

**Figure 3:**
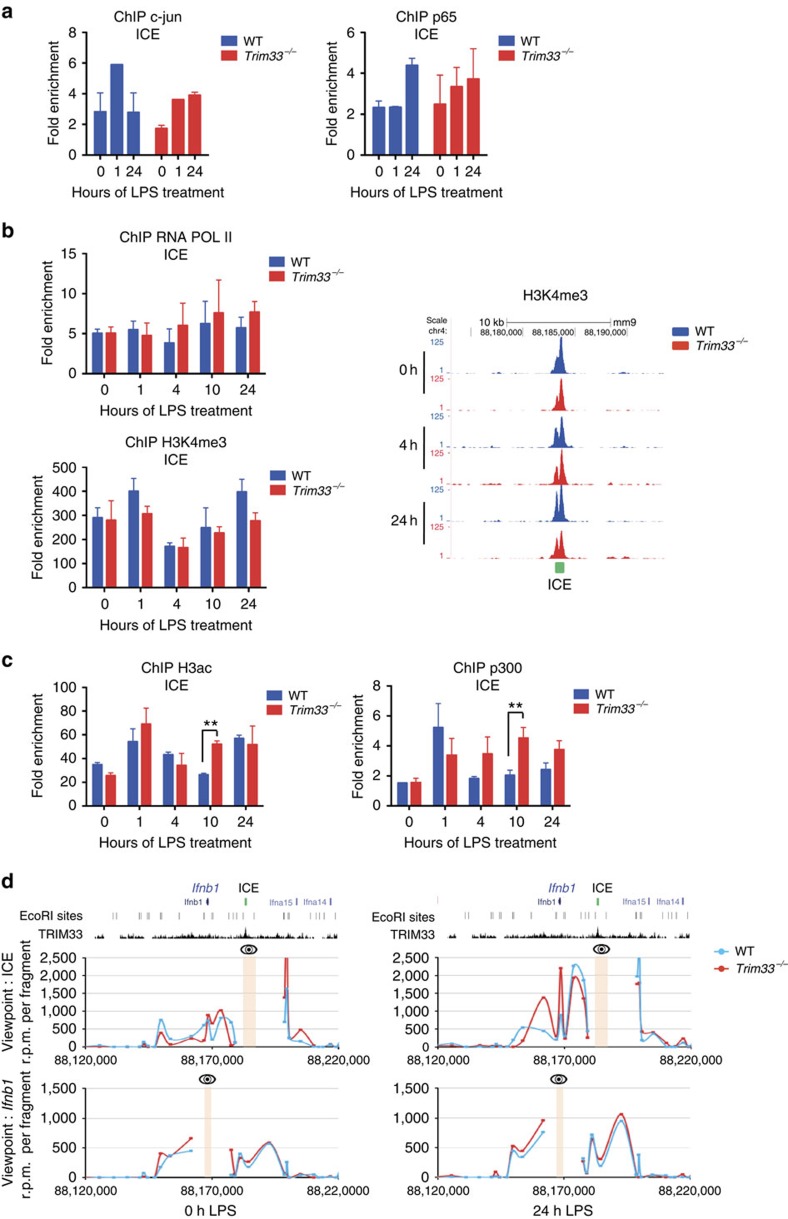
ICE chromatin structure and interaction with the *Ifnb1* promoter during activation of macrophages. (**a**) ChIP-qPCR analysis of c-jun or p65 binding at ICE in WT and *Trim33*^*−/−*^ BMDM treated for the indicated times with LPS. Mean±s.e.m., *n*=2 to 4. (**b**) ChIP-qPCR analysis of RNA Pol II (upper left panel) and H3K4me3 (lower left panel) at ICE in WT and *Trim33*^−/−^ BMDM treated for the indicated times with LPS. Mean±s.e.m., *n*=3 to 5. (Right panel) UCSC genome browser images showing H3K4me3 ChIP-seq profiles at ICE in WT and *Trim33*^*−/−*^ BMDM treated for the indicated times with LPS. (**c**) ChIP-qPCR analysis of acetylated histone H3 (left panel) and CBP/p300 (right panel) at ICE in WT and *Trim33*^−/−^ BMDM treated for the indicated times with LPS. Mean±s.e.m., *n*=4. **P<0.01, Mann–Whitney test. (**d**) DNA looping at the *Ifnb1 locus* was determined by 3C-seq performed before or 24 h after LPS activation of WT and *Trim33*^*−/−*^ BMDM using either ICE (upper panel) or *Ifnb1* gene (lower panel) as viewpoints (shown by an eye and a yellow band). Data represent normalized reads per million (r.p.m.) per restriction fragment. The *x* axis shows the genomic coordinates of the *Ifnb1* locus. The positions of the EcoRI restriction sites and the TRIM33 ChIP-seq profile in BMDM are indicated on the top.

Comparison of reported human and mouse ICE sequences showed 70% sequence homology around the TRIM33/PU.1 peak with consensus binding motifs for AP-1, NF-κB and IRF transcription factors (Supplementary Fig. 3a, left panel). Furthermore, c-JUN, the p65 subunit of NF-κB and IRF3, three transcription factors that form the *Ifnb1* enhanceosome and regulate its transcriptional activity, were also bound to ICE three hours after LPS activation of BMDM (Supplementary Fig. 3a, right panel; data from ref. 23). We studied a possible TRIM33 dependency of c-jun and p65 binding to ICE but found that TRIM33 deficiency did not modify the kinetics of their binding to ICE (Fig. 3a).

Analysis of ICE chromatin structure in macrophages showed that this region is characterized by high levels of H3K4me3 and H3K27ac modification, flanked by regions enriched for H3K4me1, all features of transcriptionally active promoters (Supplementary Fig. 3b, red graph, data from ref. 24). These epigenetic modifications are present in short-term haematopoietic stem cells (ST-HSCs) and in myeloid cells, but not in mature erythroid and lymphoid cells (Supplementary Fig. 3b). We studied the effect of TRIM33 deficiency on the chromatin structure of ICE in non-activated and LPS-activated BMDM to ask whether TRIM33 has a role in histone modification of this region. Before and during LPS activation of WT and *Trim33*^*−/−*^ BMDM, no significant differences in H3K4me1 levels on ICE could be detected (Supplementary Fig. 3c). ChIP and ChIP-seq analyses showed a constitutive binding of RNA Polymerase II (Pol II) and constitutively high levels of H3K4me3 on ICE (Fig. 3b). Prior to LPS activation, histone H3 acetylation (H3ac) on ICE was similar in WT and *Trim33*^*−/−*^ BMDM (Fig. 3c, left panel). During LPS activation, H3ac on ICE did not change in WT BMDM but was transiently increased 10 h after LPS activation in *Trim33*^*−/−*^ BMDM (Fig. 3c, left panel). This increased H3ac on ICE in *Trim33*^*−/−*^ BMDM was associated with transient increase of CBP/p300 histone H3 acetyl-transferase (HAT) -binding on ICE 10 h after LPS activation (Fig. 3c, right panel). Collectively, database analyses and our results suggest that, in BMDM, ICE exhibits a promoter-like chromatin signature established early during myeloid differentiation. TRIM33 deficiency did not modify ICE chromatin signature and only led to transient increases of H3ac and CBP/p300 recruitment on ICE whereas a sustained high expression of *Ifnb1* was observed in LPS-activated *Trim33*^*−/−*^ BMDM suggesting that TRIM33 bound to ICE might act at a distance.

To investigate how TRIM33 bound to ICE, that is located 15 kb upstream of the *Ifnb1* transcription unit, may regulate *Ifnb1* transcription, chromosome conformation capture experiments followed by deep sequencing (3C-seq) were performed in WT and *Trim33*^*−/−*^ BMDM before and after LPS activation. ICE (Fig. 3d, top) and the *Ifnb1* gene (Fig. 3d, bottom), including its promoter, were used as viewpoints. In WT BMDM, both viewpoints showed a loop between ICE and the *Ifnb1* gene before LPS activation and a twofold stronger interaction 24 h after LPS activation (Fig. 3d, blue curves). Interestingly, *Trim33* deletion did not significantly modify DNA looping in this region, even after LPS treatment (Fig. 3d, compare red (*Trim33*^*−/−*^) and blue (WT) curves). These results indicate that ICE interacts with the *Ifnb1* proximal region in a constitutive and TRIM33-independent manner, but that this interaction is strengthened following LPS stimulation.

### TRIM33 represses *Ifnb1* by preventing CBP/p300 recruitment

**Figure 4:**
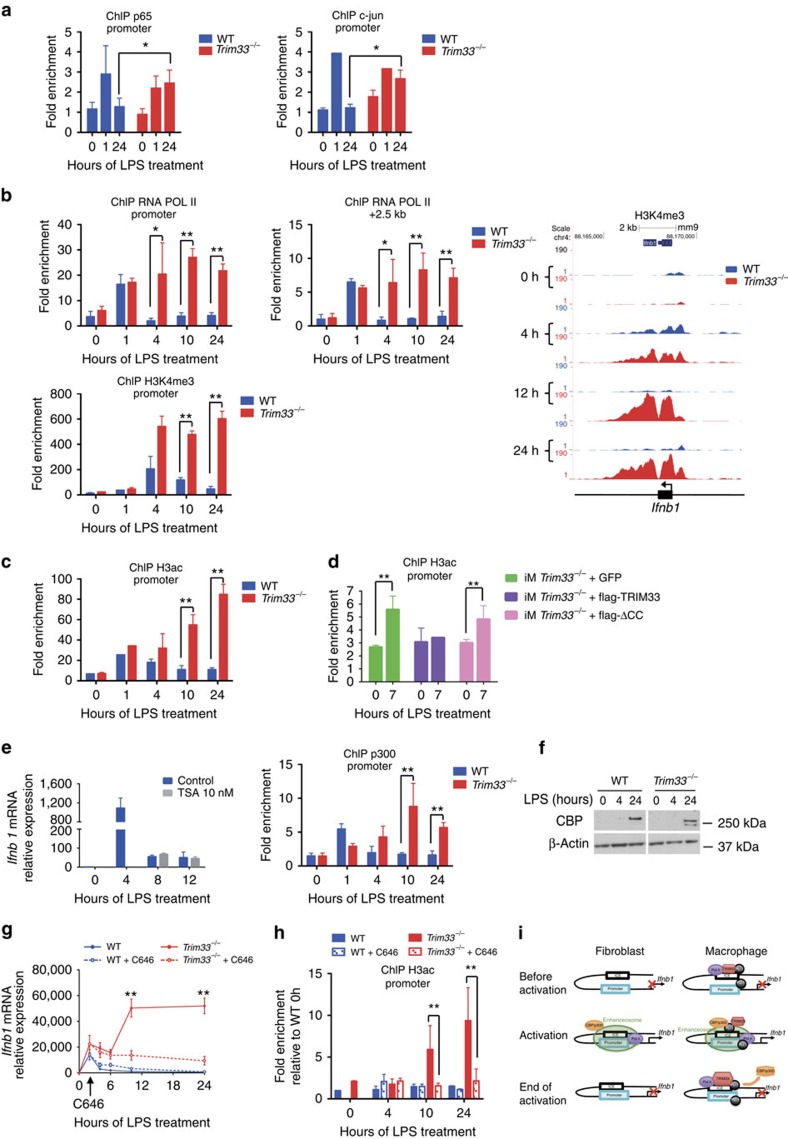
TRIM33 regulates *Ifnb1* gene expression through inhibition of CBP/p300 recruitment. (**a**) ChIP-qPCR of p65 (left panel) and c-jun (right panel) at the *Ifnb1* promoter in WT and *Trim33*^−/−^ BMDM treated with LPS. Mean±s.e.m., *n*=2 to 4. (**b**, top) ChIP-qPCR of RNA Pol II at the *Ifnb1* promoter (left panel) and at a region located +2.5 kb from TSS (middle panel) in WT and *Trim33*^−/−^ BMDM treated with LPS. Mean±s.e.m., *n*=3 to 5. (Bottom) ChIP-qPCR (left panel) and ChIP-seq (right panel) of H3K4me3 at the *Ifnb1* promoter in WT and *Trim33*^−/−^ BMDM treated with LPS. Mean±s.e.m., *n*=3. (**c**) ChIP-qPCR of H3ac at the *Ifnb1* promoter in WT and *Trim33*^−/−^ BMDM treated with LPS. Mean±s.e.m., *n*=4. (**d**) ChIP-qPCR of H3ac at the *Ifnb1* promoter in *Trim33*^−/−^ iM expressing GFP (iM *Trim33*^−/−^ +GFP), full-length flag-TRIM33 and GFP (iM *Trim33*^−/−^+flag-TRIM33), or flag-TRIM33 lacking the coiled-coil domain and GFP (iM *Trim33*^−/−^+flag-ΔCC), and treated for 0 or 7 h with LPS. Mean±s.e.m., *n*=3. (**e**, left) Relative expression of *Ifnb1* mRNA in LPS-activated WT BMDM in presence or absence of Tricostatin A (TSA), added 4 h after LPS stimulation. Mean±s.e.m., *n*=3. (Right) ChIP-qPCR of CBP/p300 binding at the *Ifnb1* promoter in WT and *Trim33*^−/−^ BMDM treated with LPS. Mean±s.e.m., *n*=4. (**f**) Western blot of CBP in WT and *Trim33*^−/−^ BMDM treated with LPS. β-actin is shown as control. *n*=4 independent experiments. (**g**) Kinetics of *Ifnb1* mRNA levels in LPS-activated WT and *Trim33*^*−/−*^ BMDM, in the presence (dotted lines) or absence (continuous lines) of C646. C646 was added 2 h after LPS stimulation and *Ifnb1* mRNA levels were determined. Mean±s.e.m., *n*=8. (**h**) ChIP-qPCR of H3ac at the *Ifnb1* promoter in LPS-activated WT and *Trim33*^−/−^ BMDM, in the presence (hatched columns) or absence (solid columns) of C646. Data are presented relative to H3ac binding in untreated WT BMDM. Mean±s.e.m., *n*=2. (**i**) Schematic representation depicting the role of TRIM33 in the macrophage-restricted *Ifnb1* gene transcription shut down. *P<0.05 and **P<0.01, Mann–Whitney test.

To characterize how TRIM33 might regulate *Ifnb1* transcription, we studied the effect of TRIM33 deficiency on temporal binding of p65 and c-jun to the enhanceosome as these two factors are major regulators of its activity. As anticipated, in WT BMDM, c-jun and p65 were recruited to the *Ifnb1* promoter at early times following LPS activation (1 h), but their binding returned to basal levels after 24 h (Fig. 4a). In contrast, in the absence of TRIM33, p65 and c-jun binding to the *Ifnb1* promoter was maintained even 24 h after activation (Fig. 4a). Thus, TRIM33 deficiency maintains enhanceosome loading by c-jun and p65 during the late phases of LPS activation.

We then determined the effect of TRIM33 deficiency on *Ifnb1* transcription and chromatin structure during LPS activation of BMDM. On the *Ifnb1* TSS, low levels of paused Pol II were observed before LPS stimulation in both WT and *Trim33*^*−/−*^ cells (Fig. 4b, upper left panel). In WT BMDM, Pol II occupancy at TSS and in *Ifnb1* 3′ region increased after 1 h of stimulation and then was lost at 4 h (Fig. 4b, upper panels). In *Trim33*^*−/−*^ BMDM, Pol II occupancy similarly increased after 1 h of stimulation but its levels were maintained up to 24 h (Fig. 4b, upper panels). H3K4me3 levels were transiently increased in WT BMDM after 4 h of LPS stimulation, whereas in *Trim33*^*−/−*^ BMDM, persistent and high levels of H3K4me3 were also observed up to 24 h (Fig. 4b, lower panel and right panel). These observations suggest that the paused Pol II seen in the absence of LPS treatment is used for rapid *Ifnb1* transcription immediately following LPS stimulation in WT and *Trim33*^*−/−*^ BMDM. Then, in WT BMDM, Pol II drops off and *Ifnb1* transcription ceases, whereas in *Trim33*^*−/−*^ BMDM, continued Pol II occupancy and H3K4me3 promote sustained *Ifnb1* transcription.

Prior to LPS activation, histone H3ac on the *Ifnb1* promoter was similar in WT and *Trim33*^*−/−*^ BMDM (Fig. 4c). This H3ac increased at 1 h after LPS activation of WT and *Trim33*^*−/−*^ BMDM. It then returned to basal levels in WT BMDM, whereas a 5- to 8-fold increase in H3ac was observed after 10 and 24 h in *Trim33*^*−/−*^ BMDM (Fig. 4c). H3ac on the *Ifnb1* promoter in LPS-activated *Trim33*^*−/−*^ iM mimicked that observed in *Trim33*^*−/−*^ BMDM (Fig. 4d). Expression of ectopic full-length flag-TRIM33 in *Trim33*^*−/−*^ iM led to decreased H3ac on the *Ifnb1* promoter 7 h after LPS stimulation (Fig. 4d), whereas the flag-ΔCC that could not rescue normal *Ifnb1* expression in *Trim33*^*−/−*^ iM (Fig. 1h) did not decrease H3ac on the *Ifnb1* promoter 7 h after LPS stimulation (Fig. 4d). These results indicate a link between TRIM33 and the regulation of H3ac on the *Ifnb1* promoter.

Increased H3ac on the *Ifnb1* promoter in LPS-activated *Trim33*^*−/−*^ BMDM indicated a possible role for TRIM33 in regulating the equilibrium between recruitment and/or activity of HAT and histone deacetylases (HDAC). Treatment of LPS-activated WT BMDM with trichostatine A, an inhibitor of HDACs, did not result in sustained *Ifnb1* expression, whereas it did alter expression of genes regulated by HDACs (Fig. 4e, left panel and Supplementary Fig. 4a). Conversely, the increased H3ac was found to be associated with CBP/p300 HAT binding on the *Ifnb1* promoter 10 and 24 h after LPS activation in *Trim33*^*−/−*^ BMDM (Fig. 4e, right panel). As total CBP protein levels were similar in WT and *Trim33*^*−/−*^ BMDM (Fig. 4f), these results indicated that TRIM33 deficiency promotes CBP recruitment. To determine the importance of CBP/p300 activity in the phenotype of *Trim33*^−/−^ BMDM, we used C646, a small-molecule inhibitor that efficiently competes with CBP/p300 substrates^25^. Adding C646 2 h after LPS induction did not modify *Ifnb1* expression in WT BMDM, but reversed the high sustained *Ifnb1* transcription level in *Trim33*^*−/−*^ BMDMs at both 10 and 24 h (Fig. 4g). This reversion was associated with decreased levels of H3ac (Fig. 4h) and reduced Pol II occupancy within the *Ifnb1* promoter region in *Trim33*^*−/−*^ BMDM (Supplementary Fig. 4b).

Altogether, these results show that enhanced CBP/p300 recruitment and activity at late times of activation is required for sustained *Ifnb1* expression in *Trim33*^*−/−*^ BMDM and that TRIM33 may regulate *Ifnb1* expression at late times by preventing the recruitment of CBP/p300.

## Discussion

Although IFN-α and IFN-β share the same receptor, IFN-β seems to be the major immune-suppressive IFN^26^ and macrophage-restricted synthesis of IFN-β can contribute to protective or pathological immune responses in the lung^27^. Surprisingly, despite the importance of IFN-β production by macrophages, no cis- or trans-acting factor that might confer a myeloid specificity to *Ifnb1* gene transcription has been characterized. The data presented in this study provide evidence of a previously unappreciated regulation of *Ifnb1* transcription in macrophages through a macrophage-restricted TRIM33 recruitment on a regulatory sequence, designated as ICE, located 15 kb upstream from the *Ifnb1* TSS.

ICE has an open-chromatin structure highly conserved during myeloid differentiation and characterized by Pol II occupancy and tri-methylation of H3K4, two features that are not typical marks of an enhancer or a repressor. In addition to a PU.1-binding site, ICE contains DNA-binding sites for transcriptional regulators such as IRF3, c-jun or p65 that are also part of the enhanceosome. ICE has been recently shown to interact with the *Ifnb1* promoter in human fibroblasts in the absence of any viral infection^22^. Here, we show that ICE can also interact with the *Ifnb1* promoter in non-activated macrophages. These combined findings indicate that ICE/*Ifnb1* loop is cell-type independent, stable and independent of viral or bacterial stimulation. Such enhancer or repressor loop stability has been described during development where they are associated with paused polymerase^28^ and in human fibroblasts where TNF-α-responsive enhancers contact their target promoters before TNF-α activation^29^.

Transient transfection in fibroblast cell lines shows that ICE acts as an enhancer of *Ifnb1* promoter activity, but only after viral infection^22^. In contrast, in macrophages, ICE acts as a repressor of *Ifnb1* promoter activity during LPS activation. Furthermore, the high and sustained expression of *Ifnb1* in LPS- or poly(I:C)-activated RAW cells where both alleles of ICE are deleted using lentiviral delivery of Cas9 and gRNA indicates that ICE has a repressor activity when these cells are stimulated. Although the lentiviral system used might increase the frequency of off-target effects^30^,^31^, the similar results obtained with three different clones and with different gRNAs make off-target effects less likely. Use of recombinant Cas9 protein^32^ or highly efficient transient transfection of RAW cells will reduce off-target effects and definitively show that ICE is a unique element that acts as an enhancer in fibroblasts, but displays promoter marks and acts as a repressor at late stages of LPS activation of macrophages. These opposite roles of ICE in fibroblasts and macrophages might be linked to recruitment of distinct proteins in the two cell types. Indeed, PU.1, which is not expressed in fibroblasts, is bound to ICE in macrophages and might be involved in the cell-specific function of ICE. Ectopic PU.1 expression in TRIM33^+^ and TRIM33^*−*^ NIH3T3 cells indicates that TRIM33-mediated repression of *Ifnb1* is dependent on PU.1 expression. However, in fibroblasts activated with poly(I:C), PU.1 expression associated with TRIM33 deficiency does not result in the sustained *Ifnb1* expression observed in poly(I:C)-activated *Trim33*^*−/−*^ BMDM. This result suggests that factors present in BMDM, but not in fibroblasts, are required for *Ifnb1* repression during the late stages of BMDM activation. Amongst haematopoietic-restricted PU.1 partners, IRF8 and IRF3 that regulate *Ifnb1* activation in BMDM^6^,^33^ are good candidates to cooperate with TRIM33 for *Ifnb1* repression during the late stages of BMDM activation.

Several TRIM proteins regulate production and action of IFN-β during innate immune response. TRIM6 is a positive regulator of IFN-β and is critical for IFN-β-mediated antiviral response^34^, TRIM25 is essential for RIG-I-mediated-type I IFN induction^35^, TRIM21 regulates IRF3-dependent IFN-β expression^36^ and TRIM56 modulates STING and subsequent IFN-β induction^37^. Here, we show the implication of TRIM33 in the transcriptional regulation of *Ifnb1*. Interestingly, TRIM33 deficiency did not alter the initial activation of *Ifnb1* transcription after LPS activation of macrophages but impaired repression of *Ifnb1* expression during the late stages of this activation. Together with the continuous binding of TRIM33 on ICE, the complementation of the *Ifnb1* phenotype in macrophages by re-expression of TRIM33 in *Trim33*^*−/−*^ immortalized macrophages suggested a direct role of TRIM33 in *Ifnb1* transcription and indicated that the activity and/or substrate availability of TRIM33 regulated *Ifnb1* expression at the end of LPS activation of macrophages.

Two lines of evidence suggest that, during the late stages of LPS activation of BMDM, TRIM33 represses *Ifnb1* expression through modulation of CBP/p300 recruitment and/or activity at the *Ifnb1* promoter. First, TRIM33 inactivation led to recruitment of CBP/p300 at the *Ifnb1* promoter and enhanced H3ac. Second, CBP/p300 activity is critical for enhanced *Ifnb1* transcription at late stages as chemical inhibition of CBP/p300 enzymatic activity could restore the regulated expression of *Ifnb1* gene in *Trim33*^*−/−*^ macrophages. As CBP/p300 acts as a switch to initially turn on *Ifnb1* gene transcription^38^, these results indicated that TRIM33 might turn off *Ifnb1* transcription during the late stages of LPS activation of macrophages by impairing the CBP/p300 recruitment and activity at the *Ifnb1* promoter. This TRIM33-dependent recruitment and activity of CBP/p300 could be direct and/or mediated by trans-acting factors such as p65 that are, in *Trim33*^*−/−*^ BMDM, loaded onto the enhanceosome during the late phases of LPS activation. However, as PU.1 can interact with CBP/p300 (refs 39, 40) and TRIM33, we propose a model where CBP/p300 and TRIM33 compete for PU.1 binding during LPS activation of BMDM. During the initial stages of LPS activation, CBP/p300 recruited by the enhanceosome might activate *Ifnb1* transcription, whereas during the late stages of LPS activation, TRIM33 might interact with PU.1, impair PU.1/CBP/p300 interaction and shut down *Ifnb1* transcription (Fig. 4i).

Numerous studies have shown the complexity of *Ifnb1* gene transcriptional regulation. Our data describe a new regulatory layer through identification of cis- and trans-acting elements that regulate *Ifnb1* gene transcription at specific steps of macrophage activation, but not in fibroblasts. These findings not only provide important insights into the cell-specific regulation of *Ifnb1* gene transcription but also extend the role of TRIM proteins as critical regulators of the innate immune response.

## Methods

### Mice

To generate deletion of *Trim33* in mature myeloid cells, *Trim*33^fl/fl^ C57Bl/6-CD45.2 mice^15^ were crossed with Lysozyme-Cre C57Bl/6-CD45.2 mice (strain name: B6.129P2-Lyz2t^m1(cre)Ifo^/J, The Jackson Laboratory). Male and female mice of 8–12 months of age were used. Experiments were performed in compliance with European legislation and with the Ethics Committee of the French Ministry of Agriculture (Agreement B9203202).

### Cell culture

For BMDM generation, mouse bone marrow (BM) was flushed out of the tibiae, femora and humeri using a syringe with PBS, filtered through a 70-μm mesh filter (Becton Dickinson) to remove debris and pelleted by centrifugation. Adherent cells were removed by incubating BM cells on culture dishes for 3 h in IMDM supplemented with 10% foetal calf serum (FCS), 1% penicillin/streptomycin (PS; Life Technologies) and 10 μM thioglycerol (Sigma Aldrich), then non-adherent cells were cultured for 7 days on culture dishes in the same medium supplemented with 25 ng ml^−1^ mouse CSF1 (Miltenyi Biotec). On day 7, BMDM were activated with LPS or poly(I:C) in IMDM supplemented with 2.5% FCS and 1% PS. For PMs, cells were collected with cold PBS and cultured for 2 h in RPMI 1640 medium (Life Technologies), supplemented with 10% FCS. All adherent cells expressed F4/80 and were considered as PMs. PM were activated with LPS or poly(I:C) in RPMI 1640 medium supplemented with 2.5% FCS and 1% PS. WT and *Trim33*^*−/−*^ iM were generated as previously described^21^. Briefly, BM cells isolated from WT and *Trim33*^*−/−*^ mice were cultured in IMDM supplemented with 10% FCS and 50 ng ml^−1^ mouse CSF1 (Peprotech). After overnight culture, the cells were transduced with a lentiviral vector expressing the SV40 large T antigen (SFFV.Tag LV). Cells were then cultured in a chemically defined medium (SFM-macrophage medium, Life Technologies) supplemented with CSF1 (50 ng ml^−1^). Single clones were isolated that expressed F4/80, CD11b but not Gr1. The resulting clones, termed iM, were maintained in IMDM supplemented with 20% foetal bovine serum, 1% PS and 50 ng ml^−1^ CSF1. iM were activated with LPS in IMDM supplemented with 2.5% FCS and 1% PS. For cell lines, RAW 264.7 and NIH3T3 cells were grown in DMEM (Life Technologies) supplemented with 10% FCS and 1% PS. RAW 264.7 cells were activated with LPS in DMEM supplemented with 1% FCS. Unless otherwise indicated, LPS (Sigma Aldrich) was used at 100 ng ml^−1^, poly(I:C) at 30 μg ml^−1^, TSA (Sigma Aldrich) at 10 nM, and C646 (Merck Millipore) at 10 μM. NIH3T3 were transfected with 10 μg ml^−1^ poly(I:C) (Sigma Aldrich).

### Chromatin Immunoprecipitation (ChIP)

Cells were fixed with 1% formaldehyde for 10 min at 37 °C, lysed in SDS lysis buffer (50 mM Tris pH8, 10 mM EDTA, 1% SDS, protease inhibitor cocktail (Roche)) and sonicated. Supernatant was diluted 10 times in IP dilution buffer (16.7 mM Tris pH8, 167 mM NaCl, 1.2 mM EDTA, 1.1% TritonX-100, 0.01% SDS, protease inhibitor cocktail (Roche)) and immunoprecipitations were carried out overnight with specific antibodies. Immunoprecipitated chromatin was collected using Protein A Agarose/Salmon Sperm DNA beads (Millipore) and, after washing and elution, reverse cross-linking was carried out with 0.2 M NaCl at 65 °C overnight. The chromatin was then digested by 20 μg of Proteinase K (Invitrogen) for 1 h at 45 °C and isolated by phenol–chloroform extraction. PCR reactions were performed using SYBR Green PCR Master Mix (Applied Biosystems) and specific primers sequences are listed below.

For sequencing, 10 ng of purified DNA from ChIP was adapter-ligated, PCR amplified and sequenced on the Illumina Genome Analyzer IIx as single-end 50 base reads following Illumina's instructions. Sequence reads were mapped to reference genome mm9/NCBI37 using Bowtie v0.12.7.

### 3C-seq

3C-seq was performed as previously described^41^, using EcoRI as first cutter and DpnII as second cutter. Briefly, cells were fixed with 2% formaldehyde for 10 min at room temperature, and nuclei were prepared by lysing cells in 10 mM Tris pH8, 10 mM NaCl, 0.2% NP-40. Nuclei were lyzed in 0.5 ml 1 × EcoRI buffer supplemented with 0.3% SDS for 1 h at 37 °C. Triton X-100 was added at 2% final for another 1 h at 37 °C. DNA was then digested with 400 units of EcoRI overnight at 37 °C. Samples were incubated for 25 min at 65 °C with 1.6% SDS, diluted to 7 ml with ligase buffer, supplemented with 1% Triton X-100 and incubated at 37 °C for 1 h. Ligation was performed for 4 h at 16 °C with 100 u T4 DNA ligase, then 300 μg proteinase K were added and samples were decrosslinked overnight at 65 °C. RNase (300 μg) was added for 45 min at 37 °C, and samples were extracted with phenol/chloroform/isoamyl alcohol (25:24:1; PCI). After precipitation, samples were digested with DpnII, extracted with PCI and precipitated, and ligated in 15 ml ligation buffer at 16 °C for 4 h before another PCI extraction and precipitation. Samples were amplified by PCR with the primers listed below, and PCR products were purified on QIAprep columns (Qiagen). The resulting libraries were sequenced on a Illumina HiSeq 2000 as single-end 100 bp reads. After trimming, reads were aligned on the mm9 version of the mouse genome using Bowtie. Results are presented as the number of reads per million reads per EcoRI fragment, in the middle of each fragment.

### Assay for IFN-β

WT and *Trim33*^*−/−*^ BMDM supernatants were collected after LPS stimulation. ELISA was performed with LegendMax Mouse IFN-β kit (Biolegend), according to manufacturer's instructions.

### Quantitative RT-PCR

Total RNA was extracted with the RNeasy Plus Micro kit (Qiagen) and reverse transcribed with random primers and Superscript III (Life Technologies). Quantitative PCR was performed using the Power SYBR green PCR master mix (Applied Biosystems) in the 7900HT Fast Real-Time or the StepOne PCR Systems (Applied Biosystems). Primer sequences are listed below.

### Lentiviral vectors for rescue and knockdown

For rescue experiments in *Trim33*^*−/−*^ iM, the lentiviral vector was constructed by inserting full-length flag-TRIM33 from the pSG5-flag-TRIM33 (ref. 15) into the pTRIP/ΔU3-MND-IRES-GFP vector, downstream the MND promoter, using the Gateway technology (Life Technologies). Deletions of the coiled-coil domain (aa 321–457), the ubiquitin ligase activity (aa 141–145) and the SMAD-interaction (aa 555–876) domain were generated by PCR. The pTRIP/ΔU3-MND-GFP was used in control experiments. NIH3T3 PU1^+^ cells were obtained by transducing a lentiviral vector pTRIP/ΔU3-MND-PU.1-IRES-GFP. For TRIM33 knocking-down in NIH3T3 or NIH3T3 PU.1^+^, the sequence of the shRNA targeting mouse *Trim33* was obtained from Sigma Aldrich (5′- CCGGCGGACTTAAATCGGTTGTTAACTCGAGTTAACAACCGATTTAAGTCCGTTTTTG -3′) and cloned in the pSuper vector (OligoEngine) downstream the H1 promoter. H1-shRNATRIM33 fragment was then cloned in the pTRIP/ΔU3-EF1α-GFP, where the GFP coding sequence is under the control of the EF1α promoter. All the constructs were verified by sequencing. Lentiviral vector production and cells transduction were carried out as described^42^. GFP-positive cells were sorted and used in functional assays.

### Luciferase assays

A 391 bp DNA fragment of murine *Ifnb1* promoter was obtained by PCR on genomic DNA and cloned into the pGL3 Basic vector (Promega) using KpnI and XhoI sites. A 408 bp DNA fragment spanning the −15 kb PU.1/TRIM33-binding site was obtained by PCR of genomic DNA and cloned upstream of the 391 bp DNA fragment of murine *Ifnb1* promoter into the pGL3 Basic vector. After sequencing of the different constructs, RAW 264.7 cells were transfected using JetPEI (PolyPlus-transfection) with the indicated constructs and the TK-renilla reporter vector (Promega), as an internal control for normalization. Twenty-four hours after transfection, cells were activated with LPS (100 ng ml^−1^) and luciferase activity was determined using the dual-luciferase reporter assay (Promega). Firefly luciferase activity was normalized with respect to renilla luciferase activity.

### Genomic deletions of ICE with the CRISPR/CAS9 system

Two guides RNA (gRNA) were designed to delete a 1,361 bp fragment containing the −15 kb region using the CRISPR Design Tool (http://tools.genome-engineering.org), to minimize the number of off-target sites. Sequences for gRNA 5A2 and 3A are 5′- TTCTCGTTCATTGTTAGCGA -3′ and 5′- CTCTAGTTTAGACGTTTAAC -3′, respectively. Singles gRNA were then cloned into the LentiCRISPRv2 (Addgene) vector, which contains the human CAS9-coding sequence, using BsmBI cloning sites. Lentivirus were produced and co-transduced into RAW 264.7 and NIH3T3. After puromycin selection (3 μg ml^−1^, Sigma Aldrich), cells were clonally isolated by fluorescence-activated cell sorting and individual clones were screened for deletion by PCR using the following primers: F5-1:5′- TGACGACAAATGTGGTACTGG -3′; F3-1:5′- CAGGCGAAAGGGAAACTAAA -3′; and R3-1:5′- TAGGAGTGGCAGATGGGAAG -3′.

Deletions of 100 or 176 bp were carried out using one gRNA guide (5′- AAACACGTTAGTTGTCAGAC -3′) flanking the PU.1/TRIM33 site at the ICE and cloned into the LentiCRISPRv2 vector as above. After puromycin selection and clonal isolation, WT and ICE^−/−^ RAW 264.7 cells were identified by PCR using the following primers: F: 5′- AGCAAAGCCGAAGAAGACAC -3′ and R: 5′- GGAAGAGGACGAGAGAACCA -3′.

All the clones were verified by sequencing of a 2 kb genomic region containing the ICE.

### Protein extraction and western blot

Total proteins from BMDM or iM were extracted using lysis buffer (50 mM Tris pH8, 300 mM NaCl, 1% Triton X-100, 0.1% sodium dodecyl sulfate, 1 mM EDTA, phosphatase inhibitor cocktail-1 and -2 (Sigma Aldrich) and complete protease inhibitor cocktail (Roche)). Proteins were separated on 3–8% Tris-Acetate gel or 4–12% Bis Tris gel (Life Technologies), transferred on a nitrocellulose membrane (Amersham) and blotted with the indicated antibodies. Following staining with HRP-coupled secondary antibodies, the proteins were detected with ECL Prime by chemiluminescence (Amersham).

### Antibodies

The following antibodies were used in western blot and ChIP: TRIM33 (3E9, Euromedex for WB (1:1,000); A301-059A, Bethyl for ChIP (1:100)), c-JUN (sc-1694X, Santa Cruz (1:200)), p65 (sc-372X, Santa Cruz (1:200)), RNA Pol II (sc-899X, Santa Cruz (1:200)), H3K4me3 (07-473, Millipore (1:500)), PU.1 (sc-352X, Santa Cruz (1:10,000 for western blot; 1:500 for ChIP)), p300 (sc-585X, Santa Cruz), AcH3 (06-599, Millipore (1:500)), H3K4me1 (ab-8895, Abcam (1:500)), Flag (M2 monoclonal, Sigma Aldrich (1:1,000)), CBP (A-22, sc-369, Santa Cruz (1:1,000)), β-actin (A5441, Sigma Aldrich (1:10,000)).

### Primer sequences

The following primer sequences were used in quantitative RT-PCR, ChIP and 3C-seq: Ifnb1 F: 5′- CTGGCTTCCATCATGAACAA -3′; Ifnb1 R: 5′- AGAGGGCTGTGGTGGAGAA -3′; Ifna F: 5′- GGATGTGACCTTCCTCAGACTC -3′; Ifna R: 5′- ACCTTCTCCTGCGGGAATCCAA -3′; Hprt F: 5′- GCTGGTGAAAAGGACCTCT -3′; Hprt R: 5′- CACAGGACTAGAACACCTGC -3′; Ifnb1 promoter F: 5′- CCACCTGTTGTTCATGATGG -3′; Ifnb1 promoter R: 5′- CATTCTCACTGCAGCCTTTG -3′; Ifnb1 ICE F: 5′- CCAAGGGTTGCGTAATGAAC -3′; Ifnb1 ICE R: 5′- CCCGATCTTCAAATCCAGTC -3′; Ifnb1 +2,5 kb F: 5′- TAGCTTCCATGCCCAGTTTC -3′; Ifnb1 +2,5 kb R: 5′- CCAAAAGTATGCCCATCACC -3′; β-globin major promoter F: 5′- GAAGCCTGATTCCGTAGAGC -3′; β-globin major promoter R: 5′- CAACTGATCCTACCTCACCTTATATGC -3′; ICE EcoRI primer: 5′- AATGATACGGCGACCACCGAACACTCTTTCCCTACACGACGCTCTTCCGATCT -3′ followed by a 4nt tag and 5′- GATGGCACACCTTGAATTC -3′; ICE DpnII primer: 5′- CAAGCAGAAGACGGCATACGAGACGACAAATGTGGTACTG -3′*; Ifnb1* EcoRI primer: 5′- AATGATACGGCGACCACCGAACACTCTTTCCCTACACGACGCTCTTCCGATCT -3′ followed by a 4nt tag and 5′- GGGTGTTCTATTTTATCAATAC -3′*; Ifnb1* DpnII primer: 5′- CAAGCAGAAGACGGCATACGATTTCTAGAGTTTCCGACTCTG -3′.

## Additional information

**Accession codes:** High throughput sequencing data have been deposited in the Gene Expression Omnibus (GEO) database (www.ncbi.nlm.nih.gov/geo), under accession codes GSE73322 (3c-seq) and GSE43654 (ChIP-seq).

**How to cite this article:** Ferri, F. *et al*. TRIM33 switches off *Ifnb1* gene transcription during the late phase of macrophage activation. *Nat. Commun.* 6:8900 doi: 10.1038/ncomms9900 (2015).

## Supplementary Material

Supplementary Information Supplementary Figures 1-4 and Supplementary References
