# Supplementary material for: TRIM33 switches off Ifnb1 gene transcription during the late phase of macrophage activation
Source: Nat Commun. 2015 Nov 23;6:8900. doi: 10.1038/ncomms9900 (PMC4673826; doi:10.1038/ncomms9900)
Supplement: Supplementary Information — Supplementary Figures 1-4 and Supplementary References [file ncomms9900-s1.pdf]

Supplementary figures

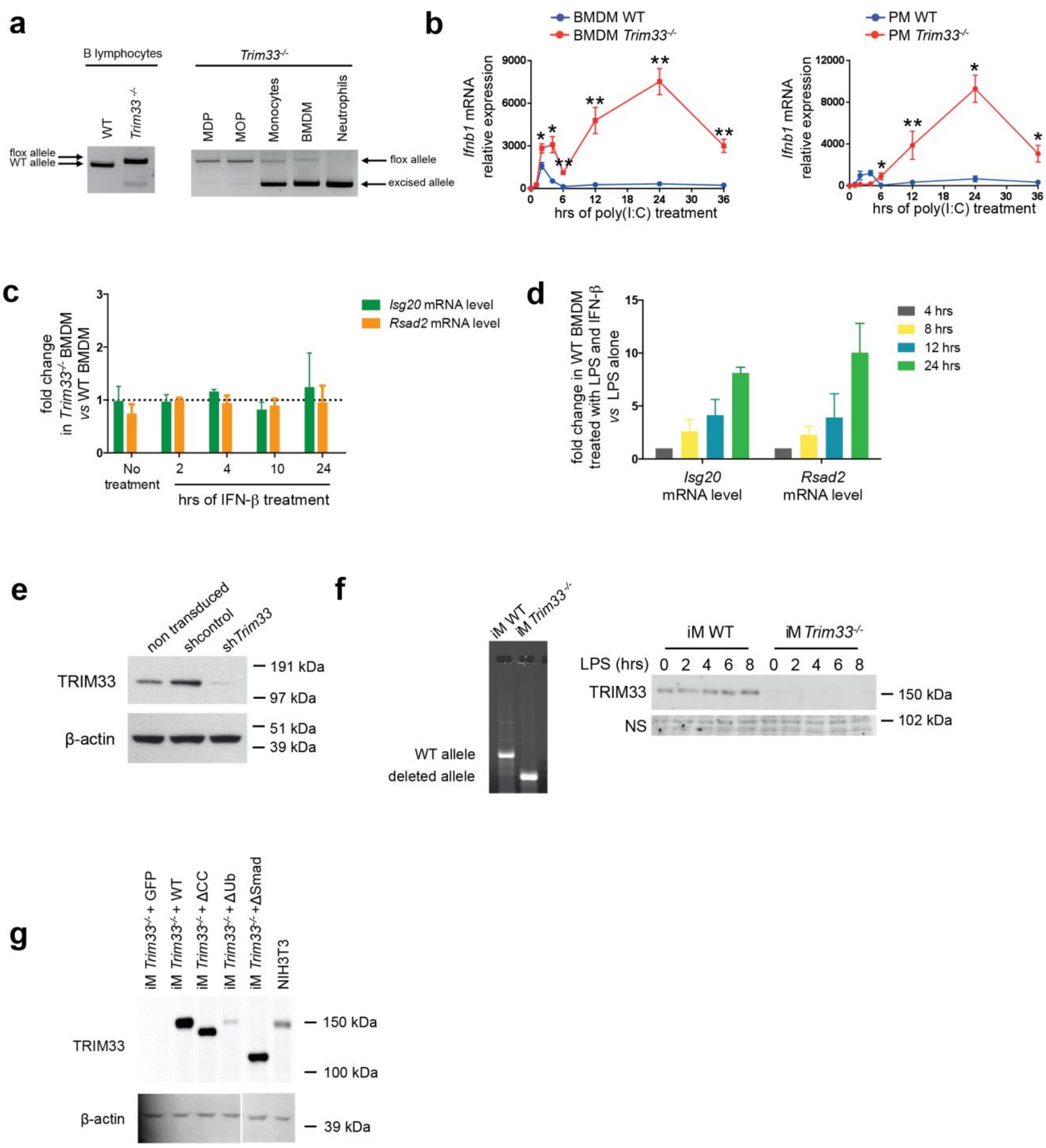

Supplementary Figure 1: Characterization of *Trim33*$^{-/-}$ macrophages

a) Genomic PCR detection of *Trim33* in B lymphocytes from WT and *Trim33*$^{-/-}$ mice (left panel) and in myeloid populations from *Trim33*$^{-/-}$ mice (right panel) (MDP: Monocyte-Macrophage dendritic cells progenitors and MOP: common Monocyte progenitors).

b) Kinetics of *Ifnb1* mRNA levels after poly(I:C) activation of WT and *Trim33*$^{-/-}$ BMDM (left panel) or PM (right panel). Mean ± SEM, n=3. * p<0.05 and ** p<0.01, Mann & Whitney test

c) Relative mRNA levels of *Isg20* and *Rsad2* in WT and *Trim33*$^{-/-}$ BMDM treated for the indicated times with IFN-β (100 U/ml). Data are expressed as fold change of *Isg20* and *Rsad2* mRNA levels in *Trim33*$^{-/-}$ versus WT BMDM. Mean ± SEM, n=3.

d) Relative mRNA levels of *Isg20* and *Rsad2* in WT BMDM treated with LPS (0.1ng/ml) and with IFN-β (100 U/ml) added 4 hours after the LPS activation. Data are expressed as fold change of *Isg20* and *Rsad2* mRNA levels over their mRNA levels in BMDM treated only with LPS (0.1 ng/ml) at the indicated times after addition of LPS. Mean ± SEM, n=3.

e) Western blot analysis of TRIM33 expression in NIH3T3 cells not transduced or transduced with lentiviral vectors encoding shcontrol or sh*Trim33*. β-actin was used as loading control.

f) (Left panel) Genomic PCR detection of *Trim33* in WT and *Trim33*$^{-/-}$ iM. (Right panel) Western blot analysis of TRIM33 in WT and *Trim33*$^{-/-}$ iM at indicated time points after LPS stimulation.

g) Western blot analysis of TRIM33 in *Trim33*$^{-/-}$ iM transduced with lentiviral vectors encoding GFP (iM *Trim33*$^{-/-}$ + GFP), full-length flag-TRIM33 (iM *Trim33*$^{-/-}$ + WT), or the indicated TRIM33 mutants. β-actin is shown as loading control.

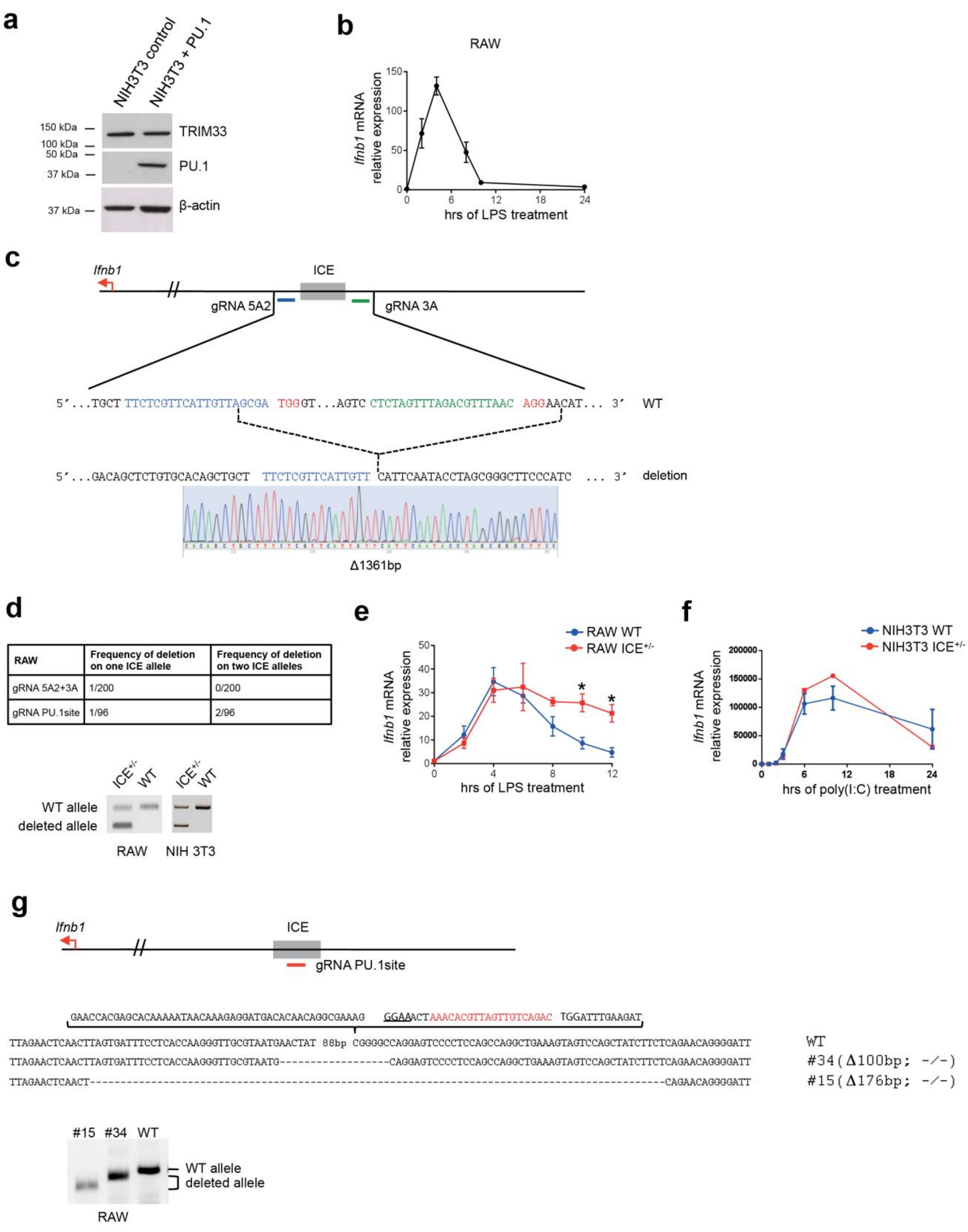

Supplementary Figure 2: Deletion of ICE with the CRISPR/Cas9 system

a) Western blot analysis of TRIM33 and PU.1 expression in NIH3T3 PU.1$^+$ cells as compared to parental NIH3T3 cells. β-actin was used as a loading control.

b) Kinetics of *Ifnb1* mRNA levels in RAW 264.7 cells activated with LPS. Mean ± SEM, n=3.

c) Schema illustrating CRISPR/CAS9-mediated deletion at the mouse ICE in the *Ifnb1* locus using two guides RNA (gRNA 5A2, blue and gRNA 3A, green). Sequences of WT and deleted alleles are shown; gRNA target sites are in blue and green, and protospacer adjacent motif (PAM) in red. Deletion junction was verified by sequencing.

d) (Top) Table showing frequency of ICE deletions in RAW 264.7 cells using lentiviral delivery of Cas9 and indicated gRNA. (Bottom) Genomic PCR of clones isolated from RAW 264.7 and NIH3T3 cells transduced with the gRNA 5A2 and 3A along with the CAS9 nuclease. WT cells and cells with a monoallelic deletion of ICE ($ICE^{+/-}$) are shown.

e and **f)** Kinetics of *Ifnb1* mRNA levels in WT and $ICE^{+/-}$ RAW 264.7 cells activated with LPS (E) or in WT and $ICE^{+/-}$ NIH3T3 activated with poly(I:C) (F). Mean ± SEM, n=3. * $p<0.05$, Mann & Whitney test

g) (Top) CRISPR/CAS9-mediated deletion of the PU.1/TRIM33 site in ICE using one guide RNA (gRNA PU.1 site, red). Sequences of WT and of two clones carrying a homozygous deletion of the PU.1/TRIM33 site in ICE are shown. gRNA target sequence is in red, PAM in italic and the consensus PU.1 site in bold.

(Bottom) Genomic PCR of individual clones for WT and $ICE^{-/-}$ RAW 264.7 showing the two homozygous deletions (clone #15 and clone #34).

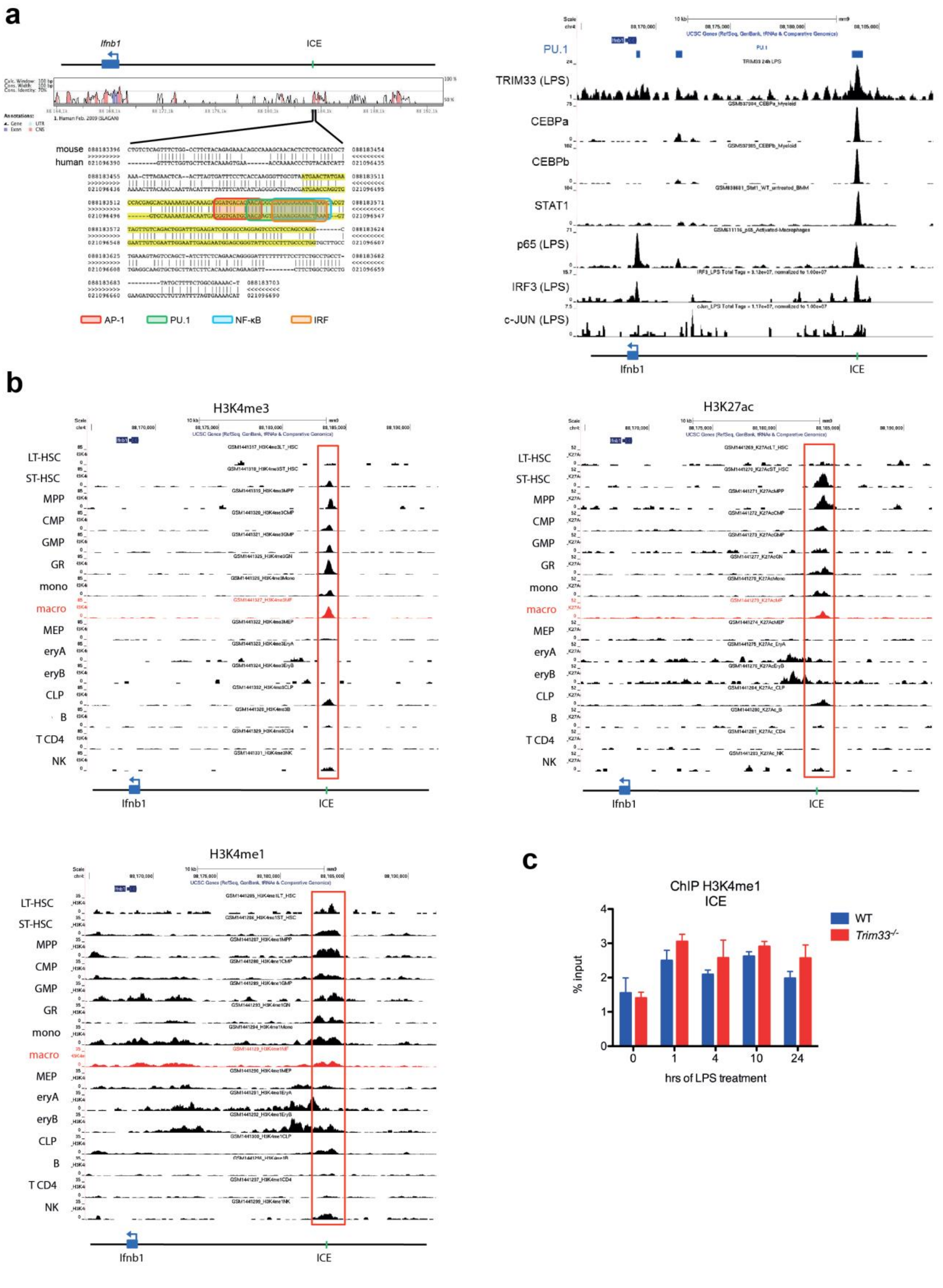

Supplementary Figure 3: Chromatin structure of ICE in hematopoietic cells

a) (Left panel) Vista plot showing conservation profile between mouse and human *Ifnb1* locus. Alignment of sequence corresponding to ICE (in yellow) and conserved

transcription factor binding sites are indicated. (Right panel) UCSC genome browser images showing the binding of the indicated transcription factor at the *Ifnb1 locus* in resting or LPS-activated BMDM (3 hours). *Ifnb1* gene and ICE are indicated. (Data sets from [1, 2, 3, 4])

b) H3K4me3, H3K27ac and H3K4me1 histone modification marks at the *Ifnb1 locus* in different hematopoietic populations. *Ifnb1* gene and ICE are indicated. Data sets from [5]. LT-HSC, long term hematopoietic stem cells; ST-HSC, short term hematopoietic stem cells; MPP, multipotent progenitor; CMP, common myeloid progenitor; GMP, granulocyte monocyte progenitor; GR, granulocytes; mono, monocyte; macro, macrophage; MEP, megakaryocyte erythroid progenitor; eryA, erythrocytes A; eryB, erythrocytes B; CLP, common lymphoid progenitor; B, B cells; T CD4, T CD4+ cells; NK, natural killer cells.

c) ChIP-qPCR data for H3K4me1 on ICE in WT and *Trim33*$^{-/-}$ BMDM treated with LPS. Mean ± SEM, n=3.

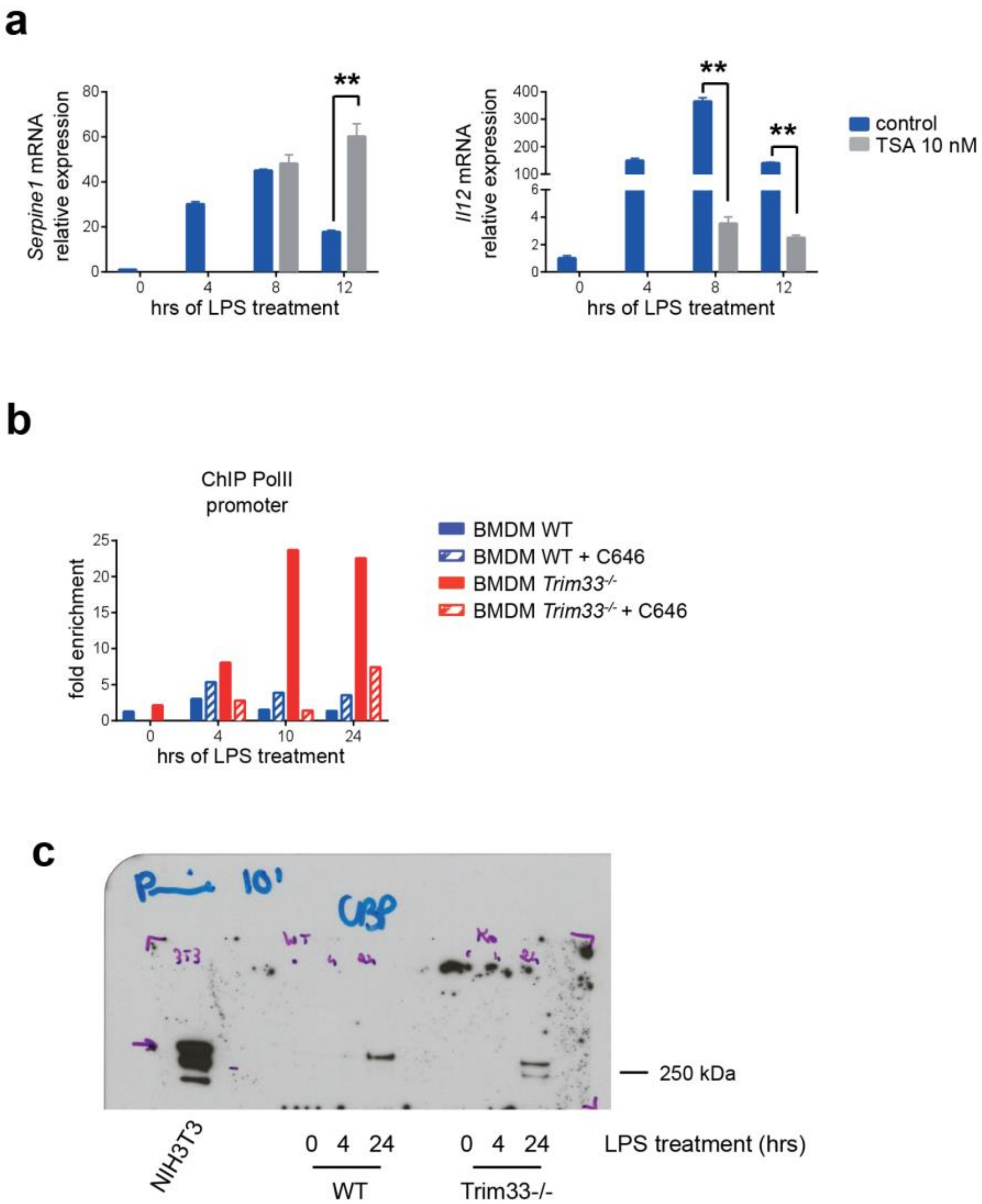

Supplementary Figure 4: Role of H3 acetylation in TRIM33 function

a) Kinetics of *Serpine1* and *Il12* mRNA levels in LPS-activated WT BMDM in presence or absence of TSA at the indicated time of LPS treatment. Mean ± SEM, n=3. ** p<0.01, Student's t test

b) ChIP-qPCR data for Pol II on *Ifnb1* promoter in LPS-activated WT and *Trim33*$^{-/-}$ BMDM, in presence (dotted columns) or absence (continuous columns) of C646, at the indicated time of LPS treatment.

c) Uncropped film corresponding to the western blot shown in Figure 4f

Supplementary References

1. Heinz S*, et al.* Simple combinations of lineage-determining transcription factors prime cis-regulatory elements required for macrophage and B cell identities. *Molecular cell* **38**, 576-589 (2010).

2. Ng SL*, et al.* IkappaB kinase epsilon (IKK(epsilon)) regulates the balance between type I and type II interferon responses. *Proc Natl Acad Sci U S A* **108**, 21170-21175 (2011).

3. Barish GD*, et al.* Bcl-6 and NF-kappaB cistromes mediate opposing regulation of the innate immune response. *Genes & development* **24**, 2760-2765 (2010).

4. Uhlenhaut NH*, et al.* Insights into negative regulation by the glucocorticoid receptor from genome-wide profiling of inflammatory cistromes. *Molecular cell* **49**, 158-171 (2013).

5. Lara-Astiaso D*, et al.* Immunogenetics. Chromatin state dynamics during blood formation. *Science* **345**, 943-949 (2014).
